# Profit Status and Employee Turnover in Iowa Nursing Homes

**DOI:** 10.1093/geroni/igab046.2179

**Published:** 2021-12-17

**Authors:** Hari Sharma, Lili Xu

**Affiliations:** 1 The University of Iowa, Iowa City, Iowa, United States; 2 University of Iowa, Iowa City, Iowa, United States

## Abstract

Employee turnover is a huge concern for nursing homes that care for millions of older individuals whose physical and cognitive impairments make them vulnerable, especially in the middle of a pandemic like COVID-19. Existing research has shown that high turnover of employees can lead to poorer quality of care. Low pay is often cited as one of the key reasons for high turnover of employees in nursing homes. For-profit nursing homes may try to maximize profits by limiting wages paid to their employees. In this study, we examine whether profit-status of a facility is associated with high turnover of its employees. We obtain data on 415 nursing homes operating in Iowa between 2013-2017. We descriptively examine the turnover trends in nurse employees and all employees over time by profit status. We evaluate whether profit status is associated with high turnover using pooled linear regressions controlling for nursing home and resident characteristics. Descriptive results show that for-profit facilities had higher turnover of nurse employees (61.1% vs. 49.6%) and all employees (56.6% vs. 45.4%). Results from multivariate regressions show that, compared to non-profit facilities, for-profit facilities had 6.93 percentage points higher (p<0.01) turnover of all employees, and 7.76 percentage points higher (p<0.01) turnover of nurse employees after controlling for facility and resident characteristics. Given existing evidence on the adverse impact of high employee turnover on nursing home quality, we need policies aimed at lowering employee turnover, targeting for-profit nursing homes.

